# Exploring RNA binding proteins in hepatocellular carcinoma: insights into mechanisms and therapeutic potential

**DOI:** 10.1186/s13046-025-03395-7

**Published:** 2025-04-24

**Authors:** Xing Ren, Wenna Yang, Xiuli Yan, Hui Zhang

**Affiliations:** 1 Institute of Interdisciplinary Integrative Medicine Research, Shanghai University of Traditional Chinese Medicine, Shanghai, 201203 China; 2 Yueyang Hospital of Integrated Traditional Chinese and Western Medicine, Shanghai University of Traditional Chinese Medicine, Shanghai, 200437 China

**Keywords:** RNA binding proteins, Hepatocellular carcinoma, Mechanisms, Therapeutic target

## Abstract

Hepatocellular carcinoma (HCC), the most prevalent type of primary liver cancer, is linked to elevated global incidence and mortality rates. Elucidating the intricate molecular pathways that drive the progression of HCC is imperative for devising targeted and effective therapeutic interventions. RNA-binding proteins (RBPs) serve as pivotal regulators of post-transcriptional processes, influencing various cellular functions. This review endeavors to provide a comprehensive analysis of the expression, function, and potential implications of RBPs in HCC. We discuss the classification and diverse roles of RBPs, with a particular focus on key RBPs implicated in HCC and their association with disease progression. Additionally, we explore the mechanisms by which RBPs contribute to HCC, including their impact on gene expression, cell proliferation, cell metastasis, angiogenesis, signaling pathways, and post-transcriptional modifications. Importantly, we examine the potential of RBPs as therapeutic targets and prognostic biomarkers, offering insights into their relevance in HCC treatment. Finally, we outline future research directions, emphasizing the need for further investigation into the functional mechanisms of RBPs and their clinical translation for personalized HCC therapy. This comprehensive review highlights the pivotal role of RBPs in HCC and their potential as novel therapeutic avenues to improve patient outcomes.

**Graphical Abstract:**

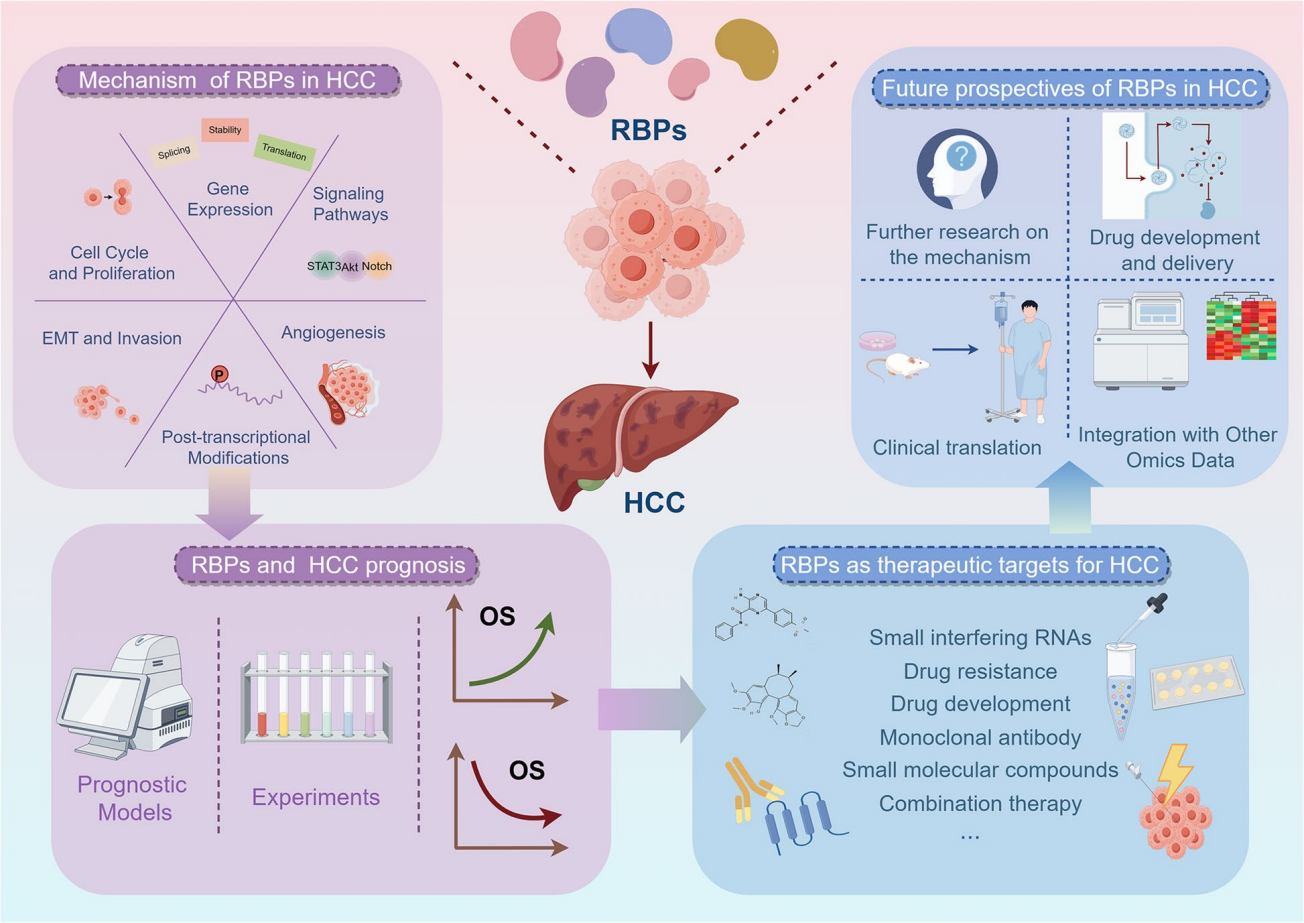

## Introduction

RNA binding proteins (RBPs) constitute a broad and heterogeneous group of posttranscriptional regulators involved in the fate of RNA, including transcription, editing, splicing, polyadenylation, transport, and turnover processes [1]. RBPs have the capacity to interact with RNA via either single or multiple globular RNA-binding domains, thereby modifying the functionality of RNA [2]. They can uphold the homeostasis of RNA metabolism by modulating the temporal, spatial, and functional dynamics of RNAs [3]. Moreover, RBPs could control the localization, stability, or translation of their target RNAs through direct interaction [4]. RBPs are highly dynamic and multifunctional regulators that exert their influence across multiple layers of post-transcriptional gene expression [5, 6]. Some RBPs not only can regulate multiple posttranscriptional processes but also function as both positive and negative post-transcriptional regulators [7]. Numerous studies have demonstrated that RBPs, as integral components of post-transcriptional RNA regulators, can lead to the onset of various diseases, including muscular atrophies, neurological disorders, and cancers [8].

Hepatocellular carcinoma (HCC) is the predominant form of primary liver cancer and represents a significant clinical challenge. It accounts for approximately 90% of all liver cancer cases [9]. HCC is the sixth most prevalent malignancy and the third leading cause of cancer-related mortality worldwide [10]. Despite the survival benefits achieved with sorafenib and regorafenib, the overall anti-tumor responses remain suboptimal [11]. Recent transcriptomics studies have demonstrated that dysregulation of the transcriptome is a driving factor in the development of HCC [12]. Dysregulated RBPs can lead to tumorigenesis by affecting gene expression [13]. Currently, investigations have elucidated that aberrant expression of RBPs is intricately associated with the initiation and progression of cancers [14]. Nevertheless, the precise biological functions and the underlying mechanisms of the majority of RBPs remain elusive. The identification of RBPs associated with HCC is of significant interest as it may enhance our understanding of HCC biology and potentially reveal novel therapeutic targets and prognostic biomarkers for this disease.

This review aims to provide a comprehensive overview of the role of RBPs in HCC. We will examine the expression patterns and functional implications of RBPs in HCC, with particular emphasis on their characterization as either oncogenic drivers or tumor suppressors. Additionally, we will explore how RBPs contribute to the mechanisms underlying HCC progression, such as gene regulation, cell cycle control, epithelial-mesenchymal transition (EMT), invasion, angiogenesis, signaling pathways, and post-transcriptional modifications. Furthermore, we will discuss the potential of RBPs as prognostic markers and therapeutic targets, highlighting recent advances and future directions in this field. By elucidating the complex interactions between RBPs and HCC, this review seeks to underscore the potential of RBPs as novel therapeutic avenues and to inform future research aimed at personalized treatment strategies for HCC.

## Function and interaction of RBPs in HCC

RBPs in cancer are usually classified into two main categories, promoting cancer RBPs and inhibiting cancer RBPs, based on their favorable or unfavorable effects on the disease. The interaction between RBPs and their targets, as well as between RBP and RBP, affects the progression of HCC (Table 1).

**Table 1:** The mechanism of promoting cancer RBPs and inhibiting cancer RBPs affecting HCC

RBPs	Putative targets	Expression	Biological roles	Ref
IGF2BP1	LINC01093	LINC01093 binds to IGF2BP1 and interferes with the interaction between IGF2BP1 and GLI1 mRNA	Inhibition of HCC cell proliferation and metastasis	[15]
IGF2BP1	miR- 186	MiR- 186 reduces IGF2BP1 mRNA and protein levels	Reduced HCC cell viability, proliferation, migration and clonogenicity	[16]
Otc4	survivin/signal transducer and STAT3 pathway	The absence of Oct4 can downregulate the expression of the survivin/signal transducer and STAT3 pathway	Downregulation of Oct4 significantly inhibits viability, proliferation and mobility of HCC cells	[17]
Otc4	LEF1/β-linker protein-dependent WNT signaling pathway	Overexpression of Oct4 activates the LEF1/β-linker protein-dependent WNT signaling pathway	Promote EMT and cancer stem cell like characteristics in HCC	[18]
BARD1	Akt, mTOR and MMP- 9	Silencing of BARD1 decreases levels of Akt, mTOR and MMP- 9 and inhibits phosphorylation of Akt and mTOR	Inhibition of proliferation, invasion and migration of HCC cells	[19]
CLDN1	miR- 29a	MiR- 29a can downregulate CLDN1 expression	Inhibition of HCC growth and migration	[20]
RBM38	HOTIAR	HOTIAR can inhibit RBM38 expression	Promote migration and invasion of HCC cells	[21]
RBM38	mdm2 and p53	Inhibition of mdm2 expression and restoration of wild type p53 expression	Inhibition of HCC proliferation and colonisation and inhibition of HCC tumourigenicity in vivo	[22]
FUS	LINC00659 and SLC10 A1	LINC00659 recruits FUS to positively regulate SLC10 A1 expression	Inhibited proliferation, migration and aerobic glycolysis glycolysis of HCC cells	[23]
FUS	LATS1/2	FUS promotes the expression of LATS/2	Inhibition of HCC progression	[24]
PDCD4	miR- 93	MiR- 93 downregulates the expression of PDCD4	Promote HCC cell proliferation	[25]
PDCD4	miR- 182	MiR- 182 negatively regulates the expression of PDCD4	Promote HCC cell migration	[26]
SORBS2	RORA	SORBS2 reduces RORA mRNA degradation	Inhibition of proliferation, invasion, migration and EMT of HCC cells	[27]

### RBPs identified as oncogenes in HCC

Several RBPs have been identified as oncogenes in HCC. These RBPs contribute to tumor progression by regulating key cellular processes such as gene expression, cell proliferation, and apoptosis. The insulin‐like growth factor 2 mRNA‐binding protein 1 (IGF2BP1) is a constituent of IGF2BP family, which also contains IGF2BP2 and IGF2BP3 [28]. IGF2BP1 is a multifunctional RBP and has been shown to be an important pro-tumorigenic factor in HCC [29]. LINC01093 can suppress HCC progression by interaction with IGF2BP1 to promote the degradation of glioma-associated oncogene homolog 1 (GLI1) mRNA [15]. In addition, miR- 186 has been identified as a possible upstream regulator of IGF2BP1. The miR- 186 mimic reduces IGF2BP1 mRNA and protein levels, which in turn inhibits the oncogenic lncRNAs H19, SNHG3, and FOXD2-AS1 and acts as a tumor suppressor in HCC [16]. Octamer‐binding transcription factor 4 (Oct4) is upregulated in various HCC cell lines and is related to overall survival and disease-free survival. The absence of Oct4 can downregulate the expression of the survivin/signal transducer and transcriptional activator 3 (STAT3) pathway, inhibiting the progression of HCC [17]. Overexpression of Oct4 activates the LEF1/β-linker protein-dependent Wnt signaling pathway to promote EMT and enhances the cancer stem cell-like characteristics of HCC cells in vitro [18]. The expression of BRCA1-associated RING Domain 1 (BARD1) in HCC tissue samples was significantly higher than that in neighboring non-cancerous liver tissues. Silencing of BARD1 led to the suppression of the proliferation, invasion, and migration of HCC cells by decreasing the levels of Akt, mTOR, and MMP- 9, as well as inhibiting the phosphorylation of Akt and mTOR [19]. Claudin- 1 (CLDN1) belongs to the transmembrane proteins and plays a crucial role in the formation of tight junctions [30]. MIR- 29a has been shown to be associated with physiological and pathological processes in tumors [31, 32] and is negatively related to CLDN1 expression in HCC. MIR- 29a can downregulate CLDN1 expression by directly binding to the 3'UTR of CLDN1, which in turn leads to inhibition of HCC growth and migration [20]. These RBPs, by promoting oncogenic processes and disrupting normal cellular homeostasis, exert pivotal influences on the development and progression of HCC. Understanding their specific mechanisms can offer insights into potential therapeutic strategies targeting these oncogenic RBPs.

### RBPs identified as tumor suppressors in HCC

In contrast to oncogenic RBPs, some RBPs function as tumor suppressors in HCC. These RBPs typically exert their suppressive effects by regulating the expression of genes involved in cell growth, differentiation, and apoptosis. RBM38, a constituent of the RNA recognition motif (RRM) family of RBPs and is expressed at a low level in HCC. HOX transcript antisense RNA (HOTAIR) is a lncRNA, and HOTIAR can promote HCC cell migration and invasion by inhibiting RBM38, suggesting that HOTIAR and RBM38 play a key role in HCC progression [21]. RBM38 destabilizes the E3 ubiquitin ligase Murine Double Minute 2 (mdm2) transcript by binding to the mdm2 3'UTR. This inhibits the expression of mdm2 and restores the expression of wild-type p53. Consequently, this results in the suppression of HCC proliferation and clonogenic capacity in vitro, as well as the attenuation of tumorigenic potential in vivo [22]. FUS (fused in sarcoma/translocated in liposarcoma) inhibits the progression of HCC through multiple mechanisms. LINC00659 Recruitment of FUS to positively regulate the expression of SLC10 A1 in HCC cells, which inhibits proliferation, migration, and aerobic glycolytic glycolysis of HCC cells [23]. The Hippo pathway is an important signal regulator of organ growth and tissue size with tumor suppressor functions [33]. LATS1/2 is one of the important components of the Hippo pathway, and FUS enhances the stability of LATS1/2, which promotes the expression of LATS/2. The FUS/LATS1/2 axis inhibits the progression of HCC through the activation of the Hippo pathway [24]. Programmed cell death 4 (PDCD4) is a tumor suppressor with reduced expression in several cancers, and PDCD4 overexpression inhibits tumorigenic [34, 35]. PDCD4 has been identified as a direct target of miR- 93, with its expression being negatively regulated by miR- 93. The downregulation of PDCD4 is implicated in facilitating the proliferation of HCC cells [25]. In addition, PDCD4 is also a direct target of miR- 182. MiR- 182 negatively regulates PDCD4 expression by targeting PDCD4 3'UTR to promote HCC cell migration [26]. Sorbin and SH3 domain-containing 2 (SORBS2) function as a tumor suppressor and is characterized by significantly diminished expression levels in HCC. Retinoic acid receptor-related orphan receptor (RORA) inhibits the progression of several solid tumors [36]. It was found that RORA is an important target of SORBS2, and SORBS2 inhibits the proliferation, invasion, migration, and EMT of HCC cells by directly binding to the 3'untranslated region (3'UTR) of RORA mRNA, thereby reducing the degradation of RORA mRNA [27].

These RBPs help maintain cellular homeostasis and suppress tumor formation. Their loss or dysfunction in HCC underscores the importance of RBPs in regulating cellular processes critical for preventing tumor progression and highlights their potential as therapeutic targets.

### The interaction of RBPs in HCC

During the development of HCC, RBPs not only interact with RNA molecules but may also interact with other RBPs, mainly in competition, supervision, and cooperation, thus affecting the progression of HCC.

RBP HuR competes with CUGBP1 for binding of E-cadherin mRNA, a critical regulator of epithelial integrity and metastasis suppression. CUGBP1 promotes E-cadherin translation and maintains cellular barrier function by enhancing mRNA stability, whereas HuR may promote cancer cell invasion by the opposite mechanism [37]. Among the RBP interactions, there is also Alternative Splicing Coupled Nonsense-mediated Decay (AS-NMD), a supervisory mechanism that enables fine regulation of gene expression by combining alternative splicing with nonsense-mediated mRNA degradation (NMD). For example, while AS-NMD is used for the inhibition of RBM10 to RBM5, RBM5 also controls the expression of RBM10 splicing variants in turn through AS-NMD to reduce the action of cancer promotion [38, 39]. In HCC patients, high expression of LIN28B was associated with poor prognosis, and its knockdown significantly inhibited tumor growth. LIN28B also forms an oncogenic fetal regulatory network of 15 RBPs that collectively drive hepatocarcinogenesis by enhancing the translational efficiency of other RBPs (e.g., IGFBP1 - 3) [40]. In addition, PTBP1 promotes HCC cell proliferation and metastasis by synergistically regulating the variable splicing of FGFR2 with other RBPs (e.g., HNRNPA1, RBFOX2, TIAL1). This splicing event activates pro-carcinogenic isoforms and enhances cell sensitivity to growth signals, thereby driving tumor development [41]. These interactions highlight the complexity of RBPs-mediated regulation of HCC, where competitive binding, cooperative splicing, and translational control have important implications for tumors. Targeting specific RBP-RBP phase interaction networks could disrupt oncogenic circuits and provide new therapeutic avenues.

## Mechanistic impact of RBPs on HCC

**Fig. 1:**
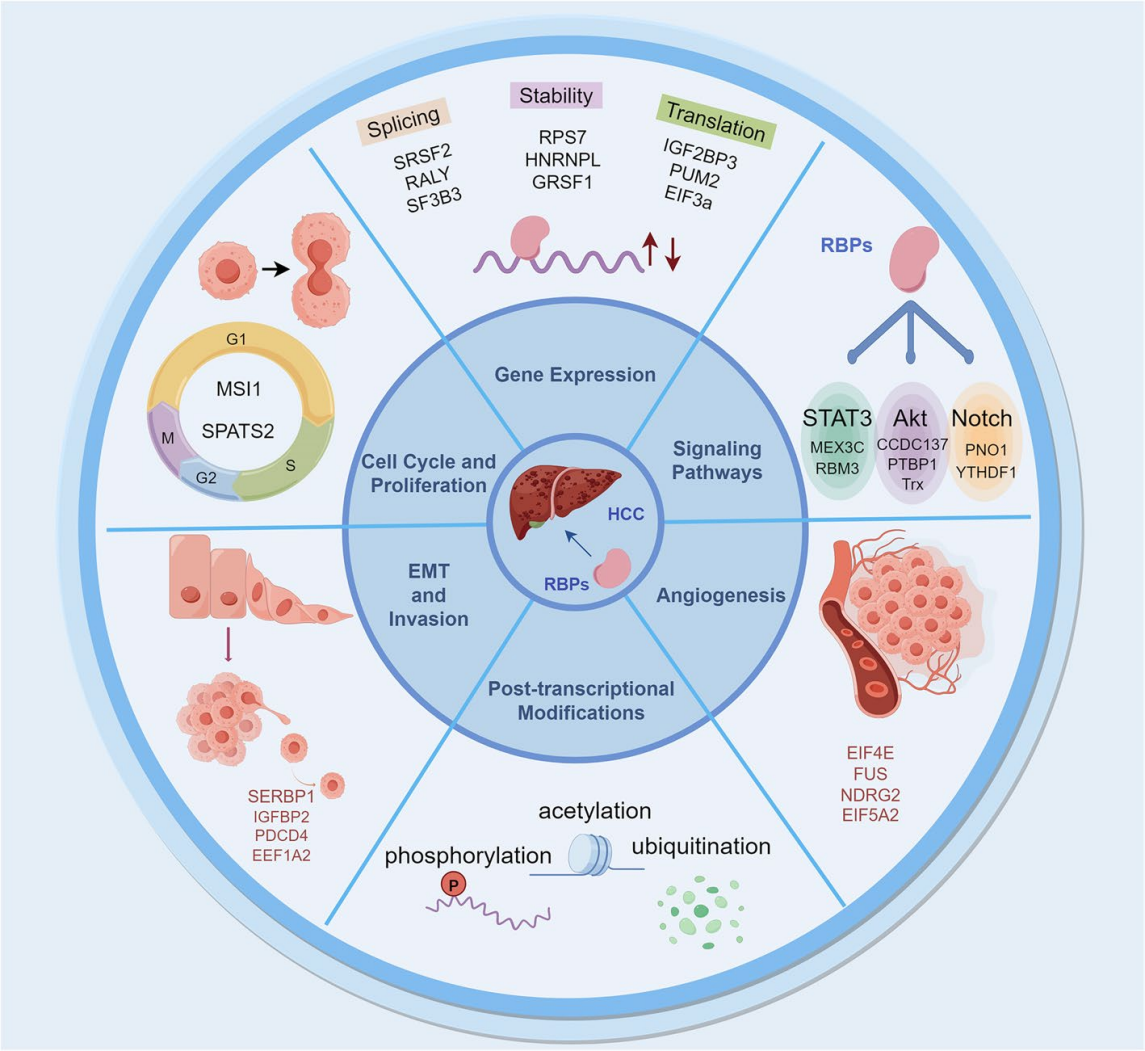
Mechanism diagram of RBPs in HCC. RBPs affect the progression of HCC by influencing gene expression, cell proliferation, cell metastasis, angiogenesis, signaling pathways, and post transcriptional modifications

RBPs play a pivotal role in HCC. They exert an impact on the progression of HCC by influencing various aspects, including gene expression, cell proliferation, cell metastasis, angiogenesis, signaling pathways, and post-transcriptional modifications (Fig. 1).

### Regulation of gene expression

RBPs play a crucial role in regulating gene expression in HCC. These proteins bind to specific RNA sequences and affect various stages of post-transcriptional regulation, such as splicing, stability, and translation. By controlling the fate and function of mRNAs, RBPs can either upregulate or downregulate the expression of key genes involved in HCC pathogenesis.

In regulating post-transcriptional splicing, serine/arginine-rich splicing factor 2 (SRSF2) can regulate splicing in HCC based on the location of pre-binding mRNA transcripts. Through alternative splicing, SRSF2 can promote the production of GCH1-L from the intron of exon 6 of GTP cyclic hydrolase 1 (GCH1), thereby promoting the development of [42]. RALY and SF3B3 synergistically regulate metastasis associated 1 (MTA1), regulate the splicing switch of MTA1 from MTA1-S to MTA1-L, leading to a decrease in MTA1-S levels, weakening the inhibitory effect of MTA1-S on cholesterol synthesis related genes and regulating the cholesterol synthesis pathway, thereby enhancing the proliferation of HCC cells [43].

In regulating RNA stability, ribosomal proteins (RPs) belong to a well-known family of classical RBPs, with human ribosomal protein S7 (RPS7) playing an important role in several cancers [44, 45]. It was found that RPS7 can stabilize LOXL2 mRNA by binding to the AUUUA motif in the 3155–3375 region of LOXL2 mRNA 3'UTR, thereby increasing LOXL2 expression by increasing the abundance of LOXL2 mRNA. Furthermore, LOXL2 could lead to the promotion of HCC cell migration and invasion by maintaining ITGB1 stability and activating the ITGB1-mediated FAK/SRC signaling pathway [46]. Recombinant S100 Calcium Binding Protein A9 (S100 A9) has been shown to be a key oncogene in HCC patients [47]. HNRNPL promotes S100 A9 mRNA stability and expression through RBP action, thereby increasing iron deposition in HCC cells [48]. G-rich sequence binding factor 1 (GRSF1) is a newly identified RBP, which has a significant impact on post-transcriptional regulation and is related to cancer progression [49]. GRSF1 and miR- 30e- 5p competitively regulate Yin-Yang 1 (YY1), a key promoter of HCC, by binding to its 3'UTR 2663–2847 region. The research uncovered the interaction relationship encompassing GRSF1, YY1, and miR- 30e- 5p. GRSF1 acts as a novel oncogenic RBP by enhancing the stability of YY1 mRNA, which provides new insights into the pathogenesis of HCC [14].

In regulating translations, IGF2BP3, an important component of the insulin-like growth factor-II mRNA-binding protein family, is highly correlated with early recurrence and poor prognosis in HCC due to its upregulation [50]. IGF2BP3 functions as an m6 A (N6-methyladenosine) reader, stabilizing target mRNAs and enhancing NRF2 translation efficiency by recognizing its m6 A modifications [51]. Pumilio homolog 2 (PUM2) directly binds to the 3'UTR of B cell translocation gene 3 (BTG3), and inhibiting PUM2 increases the mRNA and protein levels of BTG3, thereby promoting apoptosis of HCC cells [52]. Overexpression of EIF3 e, known as a proto-oncogene, leads to enhanced cellular glycolysis and a reduction in hypoxia-inducible factor 1 (HIF1a) protein. HIF1a is a transcriptional regulator of the hypoxia adaptive response. The depletion of EIF3a significantly reduced the levels of HIF1a protein and cellular glycolytic capacity. Mechanistically speaking, EIF3a regulates HIF1a protein synthesis through internal ribosome entry site (IRES)-dependent translation, which may be a potential therapeutic target for HCC [53].

### Influence on cell cycle and proliferation

RBPs exert significant influence on the cell cycle and proliferation in HCC. By regulating the expression and stability of mRNAs encoding cell cycle regulators, RBPs can modulate the transition of cells through different phases of the cell cycle. Some RBPs promote cell cycle progression by enhancing the expression of cyclins and other positive regulators, while others induce cell cycle arrest by stabilizing mRNAs encoding negative regulators. Additionally, RBPs can affect cell proliferation by regulating the expression of growth factors and their receptors, thereby controlling the signaling pathways that drive cell growth and division.

Derangement of the cell cycle is one of the drivers of tumorigenesis, and targeting cell cycle-related proteins may be an effective way of stopping tumor growth, especially critical for proliferation in specific cancer types, such as HCC [54, 55]. Musashi1 (MSI1) has been recognized as the mammalian homologue of the Drosophila protein, closely related to self-renewal and maintenance of differentiation. In recent years, many cancers have been shown to have abnormal MSI1 expression [56, 57] and can regulate cell cycle and proliferation through several signaling pathways. MSI1 prevents the G1/S phase transition of the cell cycle and promotes cell cycle progression by inhibiting the translation of p21^WAF1/CIP1^ [58, 59]. In addition, MSI1 can directly act on the mRNAs of APC and DKK1 to inhibit their translation, thereby promoting the activation of β-catenin, a key factor in the Wnt signaling pathway, and its activation further promotes the expression of downstream genes, such as c-Myc and cyclin D1, which play a key role in the G1/S transition of the cell cycle, thereby promoting the proliferation of liver cancer cells [60]. The spermatogenesis associated serine rich 2 (SPATS2) is a cytoplasmic RBP that promotes HCC progression by regulating the cell cycle [61]. In HCC, SPATS2 regulates the cell cycle through the miR- 145 - 5p/SPATS2 pathway, inhibits cell cycle arrest in the G1 phase, and promotes cell proliferation [61]. Additionally, SPATS2 regulates its methylation level through the lncRNA SNHG5, further affecting the cell cycle [62]. Despite the different mechanisms by which RBP regulates the HCC cell cycle, all have a significant impact on HCC progression.

### Influence on EMT and invasion

EMT is defined by the morphological transformation of epithelial cells, which includes the loss of apical-basal polarity and the disassembly of tight junctions, adhesive junctions, and desmosomes, resulting in impaired integrity of the cell and extracellular matrix (ECM) [63, 64]. In EMT, cell morphology also changes with the reorganization of actin cytoskeleton, and cells gain migration/invasion potential, which makes an important contribution to the progression of cancer [65, 66]. RBPs play a critical role in EMT and invasion in HCC. Certain RBPs can promote EMT by regulating the expression of EMT-inducing transcription factors and their downstream targets. These RBPs may also stabilize mRNAs encoding proteins involved in cell migration and invasion, such as matrix metalloproteinases, thereby enhancing the invasive potential of HCC cells.

Serpine mRNA binding protein 1 (SERBP1) is upregulated in HCC tissue, promoting the formation of EMT in HCC cells. CircBACH1 acts as a miR- 656 - 3p sponge, promoting the formation of EMT by increasing SERBP1 expression, thereby promoting the progression of HCC [67]. Additionally, it was found that SERBP1 is a target gene of miR- 218, which can inhibit EMT in HCC cells by targeting SERBP1 [68]. The miR- 218/SERBP1 signaling pathway can inhibit the formation of malignant phenotypes, and targeting this pathway may be a potential new pathway for HCC treatment. High expression of cell division cycle protein 45 (CDC45) is associated with poor prognosis in HCC patients. IGF2BP2 stabilizes CDC45 mRNA by m6 A modification and promotes EMT, migration and invasion of HCC cells [69]. In addition, IGF2BP2 promotes EMT by regulating key transcription factors. For example, IGF2BP1 promotes EMT by interfering with the 3'UTR degradation of LEF1 mRNA and enhancing its expression [70], and IGF2BP2 also promotes EMT by stabilizing ZEB1 mRNA [71]. PDCD4 is a tumor suppressor with reduced expression in several cancers and PDCD4 overexpression can inhibit tumorigenic [72, 73]. Studies have shown that PDCD4 can upregulate the expression of E-Cadherin while decreasing the expression of EMT markers such as N-Cadherin and Vimentin, thereby inhibiting the migration and invasion ability of tumor cells [74, 75]. Additionally, the interaction of PDCD with non-coding RNAs also has an impact on EMT and migration. For example, LncRNA miR503 host gene (miR503HG) inhibited EMT and angiogenesis in HCC via the miR- 15b/PDCD4 axis [76]. In addition, miR- 93 enhances EMT by reducing PDCD4 expression and promotes HCC invasion and metastasis [77]. The RBP EEF1 A2, as a novel transcription factor, cooperates with circ-CDYL to initiate the transcription of COL14 A1, thereby promoting ERK signaling to facilitate EMT, which may lead to HCC lung metastasis [78].

### Influence on angiogenesis

Angiogenesis is a complex and dynamic process wherein novel blood vessels emerge from pre-existing capillaries and ultimately give rise to a fully-formed and mature vascular network [79, 80]. Angiogenesis is exceptionally important for tumor progression as tumor require far more oxygen and nutrients than normal tissue [81]. Notably, HCC is one of the most vascular solid cancers and the degree of vascular infiltration is closely related to HCC growth [82]. RBPs can influence angiogenesis in HCC by regulating the expression of angiogenic factors, such as vascular endothelial growth factor (VEGF) and its receptors. By controlling the stability and translation of mRNAs encoding these factors, RBPs can modulate the angiogenic response in HCC, thereby affecting tumor vasculature and nutrient supply.

In HCC, eukaryotic translation initiation factor 4E (EIF4E) expression is elevated. The inhibition of EIF4E phosphorylation inhibits angiogenesis in human HCC [83]. EIF4E promotes tumor angiogenesis by selectively translating mRNAs closely related to angiogenesis, such as vascular endothelial growth factor (VEGF), fibronectin (FGF- 2), and matrix metalloproteinase- 9 (MMP- 9) [84, 85]. The overexpression of circ_0004018 was found to significantly inhibit angiogenesis in HCC. RBP FUS (fused in sarcomas) inhibited HCC angiogenesis by binding to ESR1 and stabilizing the expression of TIMP2, which activates circ_0004018 [86]. In addition, FUS protein regulates the expression of angiogenesis-related genes by binding to specific circRNAs. For example, FUS binding to circ_002136 led to marked reduction in miR- 138 - 5p expression, thereby derepressing SOX13 and consequently inhibiting angiogenic processes [87]. N-Myc downstream regulated gene 2 (NDRG2), belonging to the NDRG family, exerts a tumor-suppressive effect [88, 89]. VEGFA plays a key role in angiogenesis [90]. It was found that knockdown of NDRG promotes angiogenesis in HCC, which may be achieved by increasing the expression of VEGFA [91]. Besides, NDRG2 inhibits endothelial cell proliferation and tubular structure formation and reduces angiogenesis by up-regulating the expression of the oncogenes p53 and VHL, as well as down-regulating the expression of the pro-angiogenic factors HIF- 1α and VEGF [92]. Eukaryotic initiation factor 5 A2 (EIF5 A2) is frequently overexpressed in human HCC tissues. Silencing of endogenous EIF5 A2 significantly reduces MMP- 2 expression and inhibits tumor angiogenesis [93]. Additionally, the knockdown of EIF5 A2 induced shapely continuous vessel walls and well-differentiated endothelial cells, promoting the formation of a normal morphology of the vascular system [93]. RBPs related to angiogenesis are still being further explored, and targeting these RBPs may treat HCC by affecting blood supply and nutrient delivery to tumor cells.

### Modulation of signaling pathways

Aberrant activation or inhibition of signaling pathways is an important target for influencing HCC [94]. RBPs can modulate signaling pathways in HCC, which are critical for tumor development and progression. By binding to mRNAs encoding key components of signaling pathways, RBPs can regulate their expression, stability, and translation. This modulation can either activate or repress signaling cascades, leading to altered cellular responses and phenotypes.

STAT3 signaling exhibits a strong correlation with tumor growth and progression [95–97]. In multiple cancer types, the persistent activation of STAT3 is upheld through the inactivation of negative regulators, for instance, the suppressor of cytokine signaling (SOCS) [98]. Mex3 RNA-binding family member C (MEX3 C) demonstrates a marked up-regulation in metastatic HCC and is associated with poor prognosis. In HCC, MEX3 C was found to bind to the 3'UTR of SOCS3 mRNA and recruit CNOT7, thereby accelerating the degradation of SOCS3 mRNA. The downregulation of SOCS3 activates the JAK2/STAT3 signaling pathway and promotes tumor metastasis [99]. RBM3 was able to directly bind to STAT3 mRNA and stabilize its mRNA level, thereby upregulating the expression of total STAT3 and phosphorylated STAT3 (pSTAT3) and activating the STAT3 pathway, which further promoted the expression of EMT-related factors (e.g., N-calmodulin, Vimentin, etc.), leading to enhanced cell migration and invasion [100]. Multiple cancer types, including HCC, show activated Akt signaling pathway [101, 102]. AKT pathway is significantly associated with the proliferation, migration, and invasion of HCC [103, 104]. RBP CCDC137 can bind to FOXM1, JTV1, LASP1, and FLOT2 mRNAs to increase their cytoplasmic localization and thus enhance their protein expression. Elevated expression of these proteins synergistically activates AKT signaling and promotes HCC [105]. Polypyrimidine bundle binding protein 1 (PTBP1) is an oncogenic RBP associated with oncogenic splicing events. In HCC, PTBP1 expression correlates significantly with fibroblast growth factor receptor 2 (FGFR2), which promotes the transformation of FGFR2-IIIb to FGFR2-IIIc isoforms. This transition promotes EMT and activates the AKT pathways [41]. Thioredoxin (Trx) regulates angiopoietin 1 (ANGPT1) splicing by binding to LINC00152, and activation of the ANGPT1 splice heterodimer activates the PI3 K/AKT signaling pathway and up-regulates the expression of EMT-associated proteins, thereby promoting HCC migration and invasion [106]. Notch signaling is an intercellular communication mechanism that acts as a tumor promoter or tumor suppressor depending on cell type and environment [107, 108]. Previous research has also elucidated the critical role of Notch signaling in HCC [109, 110]. The RBP PNO1 assumes a key function in ribosome biogenesis, is overexpressed in HCC, and is associated with poor prognosis. Knockdown of PNO1 was shown to suppress Notch signaling by modulating the expression of Notch ligands, receptors, and downstream targets, thereby inhibiting HCC progression [111]. YTH domain-containing family protein 1(YTHDF1) can promote Notch1 gene expression by binding to the m6 A modification site of Notch1 mRNA and enhancing its stability and translational efficiency, which in turn activates the Notch signaling pathway and promotes the formation of tumor stemness and drug resistance [112].

Understanding the role of these RBP-involved signaling pathways in HCC is crucial for the development of new therapeutic strategies, which can help researchers design more precisely targeted drugs. It can also provide molecular markers for early diagnosis and prognosis assessment of HCC.

### Regulation of post-transcriptional modifications

Post-transcriptional modifications (PTMs) take various forms, including but not limited to phosphorylation, methylation, and ubiquitination. PTMs are important for gene expression [113], and abnormalities in PTMs play a key role in a variety of cancers [114, 115]. Phosphorylation serves as a critical post-translational modification that dynamically regulates the activity of key signaling molecules, thereby playing a pivotal role in tumor pathogenesis through either activation or inhibition of oncogenic pathways [116]. For example, Dnd1 binds to the 3'-UTR of LATS2, a key kinase in the Hippo pathway, thereby increasing the stability of LATS2 mRNA and its expression, leading to phosphorylation of YAP and its cytoplasmic retention. This inhibits the attenuation of EMT and suppresses HCC stem cell properties, thus exerting an anti-tumor effect [117]. It was shown that the knockdown of MEX3 A down-regulated the mRNA and protein levels of WWC1 and prolonged the half-life of WWC1 mRNA, thereby inhibiting the expression of WWC1. The down-regulation of WWC1, a negative regulator of the Hippo signaling pathway, resulted in increased phosphorylation of LATS1 and YAP1 [118]. This inhibited HCC cell proliferation and migration and increased sorafenib sensitivity.

RBPs may be involved in the regulation of histone acetylation status, affecting chromatin structure and gene expression. Histone acetylation is often associated with activated gene expression, and its aberrant regulation may lead to tumorigenesis and progression [119]. Methylation modification is the process of adding a methyl (-CH3) group to a biomolecule, usually associated with gene silencing [120]. For example, TERT can promote cancer cell growth, invasion, and metastasis in a telomere length-independent manner and is related to treatment resistance, recurrence, and poor outcomes in cancer patients [121, 122]. TERT was found to be a central regulatory factor involved in aberrant DNA methylation and AKT activation, and the TERT-DNMT3B-PTEN-AKT axis assumes a crucial function in the occurrence and development of HCC [123]. YTHDF1 promotes the stability of m6 A-modified RBM15B mRNA by binding to it, which in turn upregulates RBM15B expression and increases the overall m6 A methylation level of HCC, thereby promoting tumorigenesis and progression [124].

Ubiquitination, which encompasses both monoubiquitylation and polyubiquitination, serves as a critical post-translational modification that primarily regulates proteasome-dependent protein degradation [125]. YTH domain-containing family protein 2 (YTHDF2) increases splicing of LncFAL in an m6 A-dependent manner. LncFAL reduces iron death vulnerability by directly binding to iron death inhibitory protein 1 (FSP1) and competitively abrogating the Trim69-dependent polyubiquitination degradation of FSP1. This is an important mechanism for FSP1-dependent resistance to iron death in HCC [126]. RBP RBM39 promotes further entry of Notch2 into the nucleus by binding to LINC01977, thereby preventing ubiquitination and descending of Notch2 to promote HCC progression [127]. In short, RBPs regulate PTMs in various ways, singly or in combination. They influence intracellular protein synthesis and function, which in turn have a profound impact on the development of HCC.

## Potential roles of RBPs in HCC treatment

RBPs are gradually becoming key elements in the treatment of HCC. They hold potential application values in various aspects, including prognostic assessment, discovery of therapeutic targets, and development of novel therapeutic strategies. In the prognostic field, RBPs show promise as prognostic markers, and their differential expression in tumor and normal liver tissue correlates with disease progression and patient prognosis. In the therapeutic field, they are attractive therapeutic targets due to their role in regulating gene expression and cellular processes associated with HCC development. Understanding the potential roles of RBPs can offer new insights and approaches for the more effective treatment of HCC.

### RBPs as prognostic markers

**Fig. 2:**
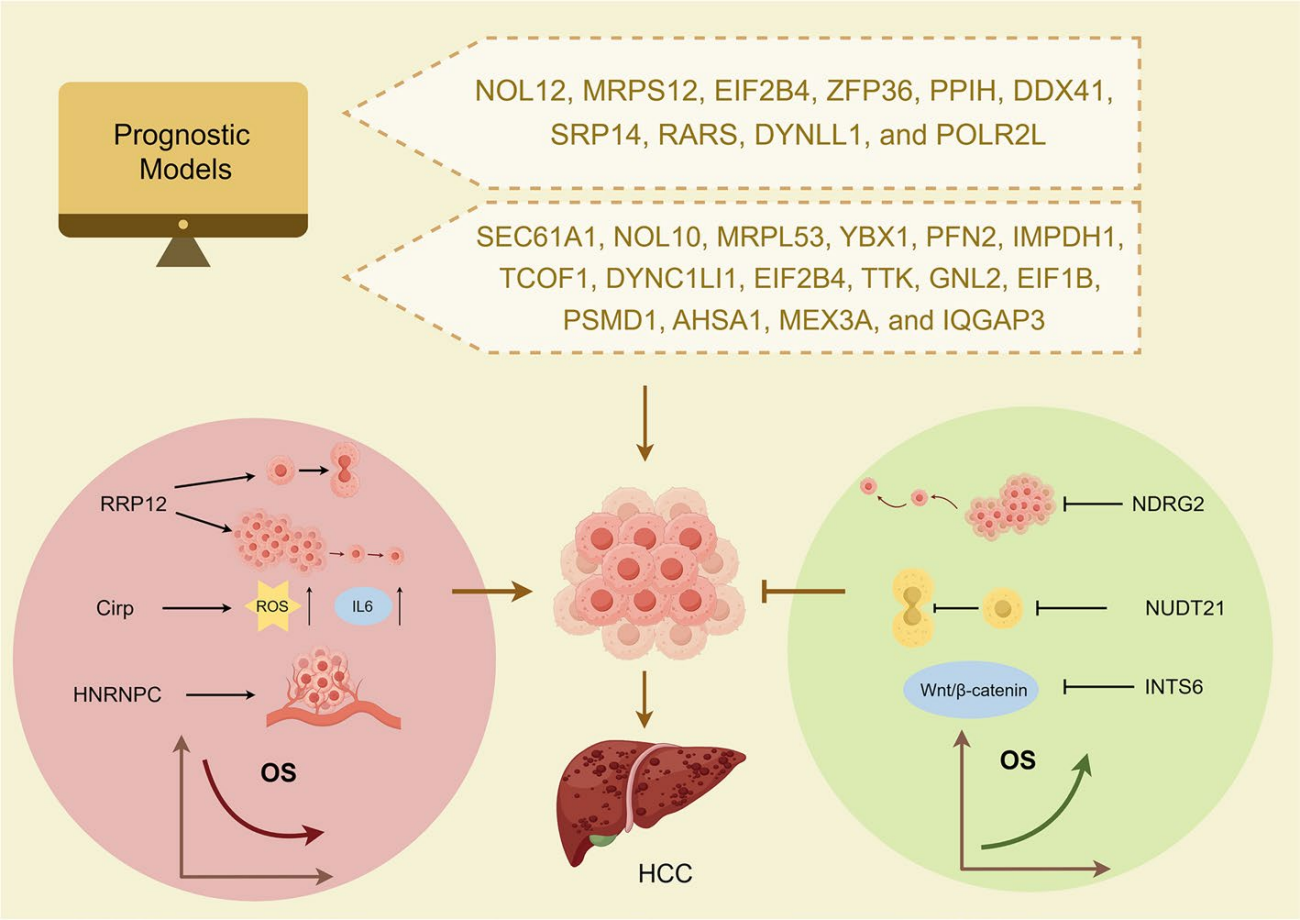
The relationship between RBPs and the prognosis of HCC. By establishing a prognostic model, a large number of RBPs that have a significant impact on the prognosis of HCC have been identified. Through experiments, it has also been found that some RBPs (such as RRP12, Crip, and HNRNPC) are positively correlated with poor prognosis of HCC through different mechanisms, while others (such as NDRG2, NUDT21, and INTS6) are positively correlated with good prognosis of HCC through different mechanisms

RBPs hold considerable promise as prognostic markers in HCC (Fig. 2). Their differential expression and activity in tumor tissues compared to normal liver tissues often correlate with disease progression, prognosis, and patient outcomes. Some RBPs are differentially expressed in cancerous and paracancerous tissues, and this differential expression is related to patient prognosis and clinical characteristics [128].

Recently, prognostic models have been widely used in medical studies and have become an important tool for determining the relationship between genes and disease prognosis by integrating multiple biological information and clinical data. A prognostic model containing 11 RBPs (NOL12, MRPS12, EIF2B4, ZFP36, PPIH, DDX41, SRP14, RARS, DYNLL1, and POLR2L) predicts the prognosis of HBV-associated HCC, including disease-free survival and progression-free interval [129]. Wang, Han et al. generated a risk score model for HCC based on TCGA and GEO databases analyzing differentially expressed RBPs between tumor and non-tumor tissues. The results showed that 16 RBPs (SEC61 A1, NOL10, MRPL53, YBX1, PFN2, IMPDH1, TCOF1, DYNC1LI1, EIF2B4, TTK, GNL2, EIF1B, PSMD1, AHSA1, MEX3 A, and IQGAP3) were identified as prognostic genes for HCC [130]. The prognostic role of several of these RBPs in HCC was again validated in other studies. By observing the tumors and adjacent normal tissues of 250 HCC patients, it was discovered that IQGAP3 positive expression was associated with poor tumor differentiation, relapse-free survival (RFS), and overall survival (OS) [131]. TCOF1 expression is abnormally elevated in HCC, regulates the EMT genes in HCC, and is related to HCC progression and poor outcome [132]. PSMD1 provides energy to HCC cells by regulating cellular lipid metabolism. It also regulates the expression of genes associated with de novo lipid synthesis through p38-JNK and AKT signaling. High PSMD1 expression is significantly associated with poor prognosis of HCC [133].

Experiments revealed that knockdown of RBP RRP12 (ribosomal RNA processing 12 homolog) inhibited the proliferation and metastasis of HCC cells. By univariate and multivariate analyses, it was concluded that RRP12 may serve as an independent prognostic maker for HCC and was significantly negatively correlated with tumor stage, tumor grade, and tumor size [134]. Cold-inducible RBP (Cirp) is increased in HCC, and knockdown of Cirp reduces interleukin- 6 production and ROS accumulation. Cirp is positively correlated with the risk of HCC recurrence [135]. Heterogeneous nuclear ribonucleoprotein C (C1/C2) (HNRNPC) is an RBP involved in nucleic acid metabolism. Beyond its established correlations with tumor size, microvascular invasion, and TNM staging, HNRNPC expression was significantly associated with an increased recurrence rate in HCC patients [136].

NDRG2 regulates CD24 expression and inhibits tumor cell invasion and migration, playing an essential role in inhibiting HCC tumor metastasis [137, 138]. NUDT21 is an interacting partner of argonaute 2 that inhibits HCC cell proliferation. Deletion of NUDT21 shortens the 3'UTRs of various oncogenes in HCC cells. These truncated 3'UTRs harbor fewer miRNA binding sites, thereby allowing oncogenes to evade miRNA-mediated regulation and become overexpressed in HCC. Consequently, this leads to unregulated proliferation of cancer cells [139]. Integrator complex subunit 6 (INTS6) is a suppressor of HCC. It can inhibit HCC growth by passing through the Wnt pathway and serve as a prognostic marker [140]. These RBPs that become prognostic markers may have important implications for the clinical management of HCC.

### RBPs as therapeutic targets

**Fig. 3:**
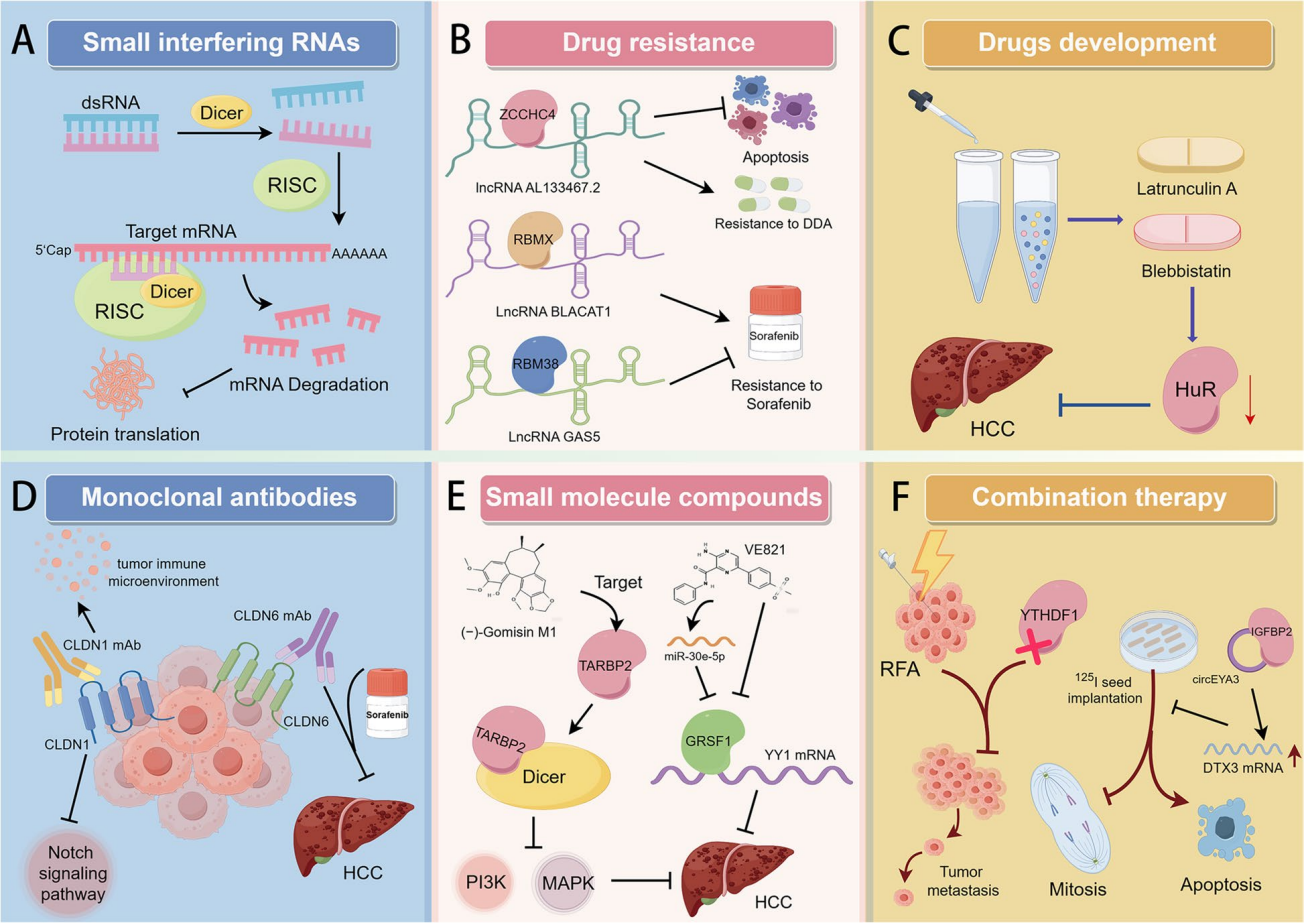
The RBPs as potential therapeutic targets for HCC. **A** Small interfering RNA: intracellular long double stranded RNA (dsRNA) is cleaved into siRNA under the action of nuclease Dicer. SiRNAs assemble with a variety of proteins to form the RNA induced silencing complex (RISC). The guide strand in RISC recognizes and binds its complementary target mRNA sequence by base complementary pairing. RISC binds to the target mRNA and cleaves the target mRNA. The ribosome cannot translate normally to produce the corresponding protein, which leads to the decrease of the expression level of the protein. **B** ZCCHC4 inhibits apoptosis signal transduction and promotes the chemical resistance of HCC cells to DDA by interacting with novel lncRNA AL133467.2. RBMX stabilizes the expression of LncBLACAT1, thus promoting the resistance of HCC to sorafenib. RBM38 reverses sorafenib resistance in HCC by binding to LncGAS5. **C** Drug research and development: cytoskeleton inhibitors latrunculin A and blebbistatin can target HuR and reduce the expression of HuR in HCC cells, thus playing an anti-cancer role. **D** CLDN1 monoclonal antibody inhibits tumor growth by regulating Notch signaling pathway and tumor immune microenvironment. Anti-CLDN6 monoclonal antibody and sorafenib have synergistic anti-HCC effect. **E** The small molecular compound VE821 significantly inhibited the expression of GRSF1/YY1 in HCC cells, and promoted the expression of miR- 30e- 5p in a dose-dependent manner. (-)-Gomisin M1 was found to target TARBP2 and promote the binding of TARBP2 to Dicer, thus inhibiting PI3 K and MAPK signaling pathways and inhibiting the proliferation, migration and invasion of HCC cells. **F** The combination therapy of radiofrequency ablation (RFA) and YTHDF1 knockout greatly inhibited the tumor metastasis induced by sublethal heat treatment. This encourages us to explore new methods and combine RFA with key intervention targets to achieve better therapeutic effects. The combination of circEYA3 and IGF2BP2 enhances the ability to stabilize DTX3L mRNA, and specifically reduces the DNA damage induced by 125I seed implantation therapy in HCC cells

RBPs represent an attractive class of therapeutic targets due to their central role in regulating gene expression and cellular processes crucial for HCC development and progression. The aberrant expression or dysregulation of RBPs can promote oncogenic processes, highlighting their potential as therapeutic targets for precision medicine in cancer. Strategies to inhibit or modulate specific RBPs could disrupt tumor-promoting pathways and restore normal cellular function. For instance, small molecules or RNA-based therapeutics, including antisense oligonucleotides or small interfering RNAs (siRNAs), could be designed to specifically target and inhibit overactive RBPs (Fig. 3A). Additionally, identifying RBPs that are critical for maintaining HCC cell viability or resistance to conventional therapies could lead to the development of novel combinatorial treatments that enhance the efficacy of existing therapies and overcome resistance mechanisms.

Chemotherapy still dominates among oncology drugs, but its resistance limits its efficacy. Tumor resistance to chemotherapy drugs remains an unresolved challenge for clinicians [141]. Recently, RBPs have gradually been recognized as an important target for improving drug resistance in tumors (Fig. 3B). Zinc finger CCHC domain-containing protein 4 (ZCCHC4) is abnormally highly expressed in a variety of cancer tissues and has been related to chemoresistance. ZCCHC4 inhibits apoptotic signaling and promotes chemoresistance to DNA damaging agents (DDA) in HCC cells by interacting with a novel lncRNA, AL133467.2 [142]. Sorafenib is a first-line agent for the treatment of HCC [143], and multiple RBPs were found to be associated with sorafenib resistance. RBMX contributes significantly to tumorigenesis and sorafenib resistance in HCC. HCC cells can improve their proliferation and colony-forming ability by stabilizing the expression of LncBLACAT1 through RBMX, thereby promoting HCC development and drug resistance [144]. In addition, RBM38 represents a new therapeutic target for sorafenib resistance through its synergistic action with LncGAS5 [145].

Drugs targeting RBP for the treatment of HCC are also in development (Fig. 3C). The cytoskeletal inhibitors latrunculin A and blebbistatin can target RBP HuR and reduce HuR expression in HCC cells to exert anticancer effects [146]. Claudins (CLDN) belong to transmembrane proteins that are important for tumor development. In vivo depletion of CLDN1 significantly diminished HCC burden, providing validation for targeting CLDN1 to prevent HCC [147]. Monoclonal antibodies (mAbs) targeting CLDN1 exhibit dual efficacy in attenuating liver fibrosis and preventing HCC, positioning them as promising therapeutic candidates for the treatment of these conditions [147]. A preclinical study also found that mAbs for CLDN1 inhibit tumor growth by regulating the Notch pathway and the tumor immune microenvironment, among other things [148]. In addition, the anti-CLDN6 mAbs showed potent anti-tumor effects and synergistic effects with sorafenib in mouse models of HCC [149] (Fig. 3D). In conclusion, RBP has great potential as a therapeutic target for HCC, and new preclinical and clinical trials are ongoing.

### Development of RBP-based therapeutic strategies

In HCC, therapeutic strategies targeting RBPs are currently under development. Among these, small-molecule-based targeted therapies are gaining increasing attention in oncology research [150] (Fig. 3E). In theory, drugs targeting specific fractions of tumors have clearer anti-cancer effects while minimizing unnecessary side effects. GRSF1 promotes HCC by enhancing YY1 mRNA stability. YY1 is a key target of miR- 30e- 5p, and GRSF1 and miR- 30e- 5p competitively regulate YY1. Using a high-throughput screening technique, the small molecule compound VE821 was found to significantly inhibit the expression of GRSF1/YY1 and promote the expression of miR- 30e- 5p in a dose-dependent manner in HCC cells. This suggests that VE821 may be a novel drug with potential to treat HCC [14]. Transactivation response element RBP2 (TARBP2), which serves as a crucial constituent of the miRNA-induced silencing complex (RISC), exhibits reduced expression levels in HCC cell lines. Moreover, it has been demonstrated that the deletion of TARBP2 can enhance the proliferation, migration, and invasion capabilities of HCC cell lines [151]. As a Dicer chaperone protein, TARBP2 can maintain miRNA production and subsequent gene silencing [152]. A number of small molecule compounds targeting TARBP2 were screened. (−)-Gomisin M1 (GM) was found to target TARBP2, affecting miRNA maturation with higher efficiency than enrofloxacin, and promoting the binding of TARBP2 to Dicer, thereby inhibiting PI3 K, MAPK signaling pathways to suppress HCC cell proliferation, migration, and invasion [153]. These results support the use of small molecule compounds targeting RBP as a novel therapeutic strategy for treating HCC.

Radiofrequency ablation (RFA) is one of the early-stage treatments endorsed by guidelines from both the American Association for the Study of Liver Diseases and the European Association for the Study of the Liver, which utilizes heat stress generated by high-frequency alternating current electricity to kill cancerous cells [154]. Despite the many advantages of this minimally invasive treatment, inadequate radiofrequency ablation (IRFA) induces sublethal heat stress, which has been shown to promote the progression of HCC [155]. It was found that knockdown of YTHDF1 greatly inhibited tumor metastasis induced by sublethal heat treatment in a mouse model of HCC [156]. This inspired us to explore new approaches that could combine both RFA and intervening key targets for better therapeutic effects (Fig. 3F).

There is also iodine- 125 (^125^I) seed implantation therapy for the treatment of HCC, which significantly reduces the proliferation, invasion, and metastasis of HCC cells by releasing large amounts of X-rays and γ-rays to cause DNA damage to the tumor cells, resulting in G2/M arrest, inhibition of mitosis, and induction of apoptosis. However, the clinical outcomes of ^125^I radiation therapy for HCC are limited [157]. It was found that circEYA3 binding to IGF2BP2 enhanced the ability to stabilize DTX3L mRNA, thereby specifically attenuating radiation-induced DNA damage in HCC cells [158]. Therefore, if IGF2BP2/DTX3 can be targeted to reduce its expression, it may increase the sensitivity of HCC to ^125^I and improve the therapeutic efficacy, which needs to be further investigated in the future (Fig. 3F). In conclusion, therapeutic strategies related to RBP are under further development, which may open new horizons for the clinical treatment of HCC.

## Future perspectives

RBPs hold great promise for the treatment and research prospects of HCC. As our understanding of RBPs in HCC continues to evolve, several key areas of focus are emerging. Further research on the mechanisms is crucial for a deeper understanding of how RBPs influence HCC at the molecular level. The advancement of innovative drug delivery systems and successful clinical translation of preclinical discoveries are critical for developing targeted RBP-based therapeutics, thereby bridging the fundamental research-clinical application gap. Additionally, incorporating RBPs into multi-omics approaches can provide a more comprehensive understanding of their roles in HCC, with the potential to uncover new therapeutic targets and insights. Exploring these future prospects of RBPs can drive innovation and progress in the treatment of HCC and other cancers.

### Mechanistic studies

**Fig. 4:**
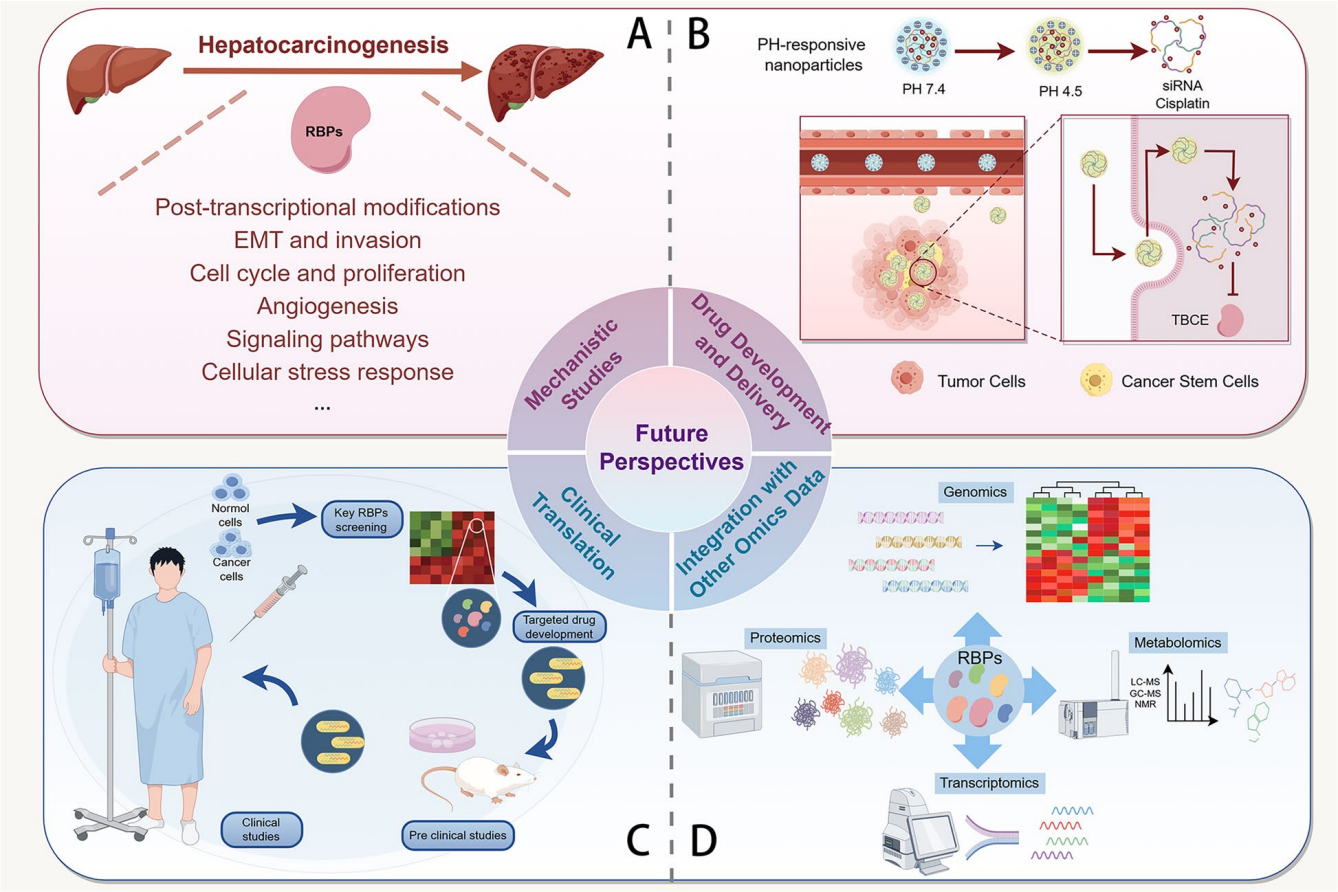
Future prospectives of RBPs in HCC. **A** The mechanism of RBPs'role in HCC still needs to be further explored in the future. **B** Drug delivery targeting RBPs is still a difficult problem, such as the delivery of PH-responsive nanoparticles targeting RBP TBCE. **C** The treatment methods related to RBPs still need a long pre-clinical research and clinical research to complete the clinical transformation process. **D** The combination of RBPs and other omics data (such as genomics, transcriptomics, proteomics and metabolomics) in the future may provide more ideas for the treatment of HCC

A more comprehensive understanding of the molecular mechanisms underlying the regulatory roles of RBPs in HCC pathogenesis is critically important (Fig. 4A). Future research should focus on elucidating the precise molecular interactions between RBPs and their target RNAs, as well as their effects on cellular pathways. RBPs are not only involved in post-transcriptional modifications of RNA but also play key roles in a variety of biological processes such as cellular stress response, metabolism, and cell adhesion [159]. For example, certain RBPs are able to regulate the expression of tumor-related genes and promote tumor formation by affecting RNA splicing and translation [160]. In HBV-associated HCC, it was discovered that there were significant alterations in the expression of mRNAs associated with RBPs, and these changes were closely related to the prognosis of the tumor [161]. In addition, by constructing a prognostic model based on RBPs-associated mRNAs, the investigators were able to identify biomarkers related to the progression of HCC, thus providing new diagnostic and therapeutic ideas for the clinic [130]. Therefore, an in-depth study of the functions of RBPs and their coordinated roles with other regulatory factors will help to reveal the molecular mechanisms of tumors such as HCC and offer a theoretical foundation for the advancement of new therapeutic approaches.

### Drug development and delivery

The development of targeted therapies against RBPs necessitates advancements in drug design and delivery systems. Researchers should aim to develop specific inhibitors that selectively modulate RBP function without affecting normal cellular processes.

Nanotechnology has opened new doors for nucleic acid-based drug delivery in the field of cancer treatment [162, 163]. PH-responsive nanoparticles are used as a material to encapsulate drugs (Fig. 4B). In the acidic environment of tumors, the polymer shell of the nanoparticles will be removed, exposing the positively charged core, which promotes the uptake of negatively charged tumor cells by the cell membrane [164, 165]. A study encapsulated siRNA and cisplatin into nanoparticles, which can target the protein TBCE and significantly inhibit the progression of HCC [166]. Additionally, novel delivery mechanisms need to be explored to ensure effective and targeted delivery of RBP-based therapies to tumor cells.

As the comprehension of RBPs and their multifaceted roles in tumorigenesis advances, targeted drug development against these regulatory molecules is rapidly emerging as a promising frontier in anticancer therapeutics. Modern systems analyses have revealed atypical structural domains of a variety of RBPs, which offer new possibilities for targeting RBP interactions [167]. In addition, oligonucleotide therapy, an emerging class of drugs, is showing potential for targeting difficult-to-target proteins and non-coding RNAs in clinical studies, which opens up new directions in the development of precision anti-cancer drugs [168]. Through these studies, drug development targeting RBPs has provided new ideas for cancer therapy. In addition, new delivery mechanisms need to be explored to ensure that RBP-based therapies are delivered to tumor cells in an effective and targeted manner.

### Clinical translation

Bridging the gap between preclinical findings and clinical application is a significant challenge (Fig. 4C). Rigorous clinical trials are required to assess the safety, efficacy, and potential side effects of RBP-targeted therapies. Furthermore, the integration of RBPs into personalized treatment regimens, based on individual patient profiles, could enhance therapeutic outcomes.

In recent years, the treatment options for HCC have been enriched. For example, for unresectable HCC, treatment options may include liver transplantation, ablation, radiation therapy, and systemic therapy [170]. Among these therapeutic approaches, the use of RBPs may provide new therapeutic opportunities for patients, especially with the collaboration of a multidisciplinary team that is able to better evaluate and adjust the therapeutic regimen [170]. Gene transfection has been put forward as a novel strategy aimed at augmenting the effectiveness of antitumor drugs, especially in the treatment of refractory or metastatic cancers. By combining gene therapy with conventional chemotherapeutic agents, studies have shown that this combination therapy can significantly improve anti-tumor effects [170]. Notably, the integration of RBPs may also be combined with other therapeutic approaches, such as sorafenib, a targeted therapeutic agent for advanced HCC, which has shown potential for prolonged survival in clinical trials [170].

Furthermore, targeting RBPs in the clinical treatment of HCC faces many issues and challenges. For example, DAZAP1 is significantly upregulated in HCC and is associated with multiple malignant features and poor patient prognosis [170]. DAZAP1 regulates the stability of SLC7 A11 mRNA by interacting with its 3'UTR, thereby affecting cellular sensitivity to chemotherapeutic agents. Given the mechanistic complexity of DAZAP1, therapeutic strategies targeting this protein require precise optimization to ensure effective functional modulation. ZCCHC4, as a tumor-promoting factor, is highly expressed in multiple cancer tissues and is associated with poor prognosis. Research has found that targeting ZCCHC4 can enhance the sensitivity of HCC cells to DNA damage drugs, thereby improving chemotherapy efficacy [142]. In summary, identifying drugs targeting key RBPS in HCC through preclinical studies not only provides new therapeutic options for patients with HCC but may also open up new avenues for the treatment of other types of cancer. Future research should continue to explore the functions of RBPs and their roles in cancer to advance the clinical application of targeted therapies.

### Integration with other omics data

Incorporating RBPs into multi-omics methodologies, including genomics, transcriptomics, proteomics, and metabolomics, enables a more comprehensive elucidation of their role in HCC (Fig. 4D). This integrative approach could reveal new insights into the interactions between RBPs and other molecular pathways, potentially uncovering additional therapeutic targets.

RBPs play key roles in gene regulation, and their functions and interactions are essential for proper cellular functioning. Through high-throughput sequencing and other genomics technologies, we were able to systematically explore novel mechanisms of RBPs in cancer development [170]. For example, recent studies have shown that cross-references between RBPs and cancer-associated genes reveal a set of 411 proteins that may be relevant to cancer biology and that are involved in a diverse array of cellular processes, including coping with stress, metabolism, and cell adhesion [159]. In addition, studies combining multi-omics data have shown that interaction-mediated control of protein abundance occupies an important position in cancer samples, suggesting a potential role for RBPs in regulating protein levels [170]. This comprehensive approach not only helps to reveal the mechanism of RBPs in cancer formation, but may also provide clues for new diagnostic and therapeutic methods [159]. In summary, combining RBPs with multi omics methods will provide us with a more comprehensive perspective and help us understand the complex roles of these key molecules in cancer and other diseases.

## Discussion and conclusion

RBP, named for its ability to bind to various RNAs (e.g., mRNAs, miRNAs, and lncRNAs, etc.), has received increasing attention for its role in HCC. Research have shown that RBPs play critical role in the genesis and development of HCC. Multiple studies have elucidated the critical involvement of RBPs in HCC, highlighting their diverse functional roles in HCC pathogenesis [170, 170]. RBPs influence HCC progression through multiple distinct molecular mechanisms. For example, abnormal expression of certain RBPs may lead to overexpression of oncogenes or inactivation of tumor suppressor genes, thereby promoting tumor growth and metastasis [130]. RBP is also inextricably linked to biological processes, including tumor cell proliferation, angiogenesis, tumor migration, post-transcriptional modifications, and activation of key signaling pathways, all of which play pivotal roles in cancer progression. In addition, the role of RBP in HCC prognosis should not be ignored. By analyzing HCC patient samples, researchers found that the expression levels of certain RBPs were closely associated with patient survival. These RBPs may become important diagnostic and prognostic markers for HCC. Based on this, an RBP-related prognostic model has been developed, capable of effectively predicting the survival outcomes of HCC patients [170, 170]. These models not only help to assess the prognosis of patients, but also lay the groundwork for developing personalized treatment protocols and provide theoretical underpinnings for subsequent targeted therapies.

With the advancement of high-throughput sequencing technology, an increasing number of RBPs associated with HCC have been identified, and therapeutic strategies targeting these RBPs are gradually being developed. For example, drug development targeting RBP has had a significant impact on inhibiting HCC and mitigating related side effects [170]. In addition, nanodrug delivery systems targeting RBP show potential applications. By designing nanocarriers specifically targeting RBP, the concentration of the drug at the tumor site can be increased, enhancing the therapeutic effect and reducing the side effects. This strategy provides a new way of thinking for the treatment of HCC, which is especially important in overcoming drug resistance.

Although numerous researchers have conducted a large number of studies on these mechanisms, the diversity and variability in how different RBPs influence HCC pathogenesis underscore the need for further investigation. Elucidating the precise molecular mechanisms by which RBPs contribute to HCC could provide critical insights into the fundamental processes driving tumor initiation and progression. Drug delivery is an important step in applying RBP-related research findings to the clinic. Current research is already exploring how to effectively deliver RBP-targeted drugs to the tumor site to improve therapeutic efficacy and reduce side effects [170]. This will require a combination of advances in nanotechnology and biomaterials science to develop more precise drug delivery systems. Future research into the clinical translation of RBP needs to be integrated with existing therapeutic strategies to assess its efficacy and safety in real-world clinical settings. Validating the potential of RBP as a therapeutic target through clinical trials could provide new treatment options for HCC patients [170]. In addition, strategies combining immunotherapy and targeted therapy may also bring new breakthroughs in the treatment of HCC [170]. Of course, due to the uncertainty of the safety and versatility of RBPs, extensive testing will be required before these therapeutic strategies can be formally utilized in clinical practice. Further additional preclinical trials targeting key RBPs for the treatment of HCC will be needed in the future. Alternatively, explorations in association with multi-omics will provide a more comprehensive view of RBP in HCC. Multi-omics analysis can integrate diverse data types, including genomics, transcriptomics, proteomics, and metabolomics, thereby facilitating the elucidation of the intricate regulatory networks and functional interactions of RBPs in HCC.

In conclusion, RBPs represent a promising frontier in HCC research, offering new insights into disease mechanisms and potential therapeutic strategies. Continued research is essential to fully exploit the therapeutic potential of RBPs and address the challenges that lie ahead. As our understanding of RBPs deepens and new technologies emerge, RBPs could become central components of more effective and personalized treatment regimens for HCC.

## Data Availability

No datasets were generated or analysed during the current study.

## Abbreviations

DDA: DNA damaging agents
ECM: Extracellular matrix
EMT: Epithelial-mesenchymal transition
mAbs: Monoclonal antibodies
OS: Overall survival
PTMs: Post-transcriptional modifications
RBPs: RNA-binding proteins
RFA: Radiofrequency ablation
RFS: Relapse-free survival
siRNAs: Small interfering RNAs
